# Drivers and impact of the 2021 extreme warm event in the tropical Angolan upwelling system

**DOI:** 10.1038/s41598-024-67569-7

**Published:** 2024-07-22

**Authors:** Rodrigue Anicet Imbol Koungue, Peter Brandt, Arthur Prigent, Léo Costa Aroucha, Joke Lübbecke, Arielle Stela N. Imbol Nkwinkwa, Marcus Dengler, Noel Keenlyside

**Affiliations:** 1 GEOMAR Helmholtz Centre for Ocean Research Kiel, Kiel, Germany; 2 Faculty of Mathematics and Natural Sciences, Kiel University, Kiel, Germany; 3 Earth System Physics, The Abdus Salam International Centre for Theoretical Physics, Trieste, Italy; 4 Geophysical Institute, University of Bergen and Bjerknes Centre for Climate Research, Bergen, Norway; 5 Nansen Environmental and Remote Sensing Centre, Bergen, Norway

**Keywords:** Climate sciences, Environmental sciences, Ocean sciences

## Abstract

Benguela Niños are extreme warm events that typically occur during the main downwelling season (austral fall) in the tropical Angolan upwelling system when the biological productivity is low. However, the extreme warm event that occurred between April and August 2021 stands out due to its late timing. It occurred and peaked during the main upwelling season in austral winter with sea surface temperature anomalies exceeding 2 °C in the Angola-Benguela area in June 2021. This led to an unprecedented reduction of the net primary production off southern Angola. Both local atmospheric processes and remote influences (via downwelling coastal trapped wave propagations) have contributed to the onset of the extreme warm event in April and its intensification towards the peak phase in June. Moreover, the poleward advection of warm equatorial waters toward the Angola-Benguela area in May 2021 might have contributed to the warming, since the transport of the Angola Current, as estimated from observations, was notably elevated, amounting to 2.1 Sv.

## Introduction

The tropical Angolan upwelling system (TAUS, 6°S–17°S) located in the southeastern tropical Atlantic hosts a highly productive marine ecosystem enabling the fishing sector in Angola to be the third most important source of income for the national economy^1–5^. Its seasonal cycle is marked by maximum productivity as well as minimum sea surface temperature (SST) in austral winter (July–August–September, JAS)^6,7^. In contrast to other eastern boundary upwelling systems, the TAUS is not primarily wind-driven, since its maximum productivity occurs when the alongshore wind stress and wind stress curl (WSC) are at their seasonal minimum^7,8^. Instead, the combined effect of poleward propagating upwelling coastal trapped waves (CTWs) and turbulent mixing induced by internal tides was found to explain the seasonal cycle of productivity in the TAUS^9,10^. A key feature in the TAUS is the warm poleward Angola Current along the Angolan coast^11^. The Angola Current and the TAUS are under the influence of CTWs either remotely forced via equatorial Kelvin waves (EKWs) impinging at the eastern boundary^4,8,12–16^ or forced between the equator and 10°S along the southwestern coast of Africa by alongshore winds^17^. The seasonal cycle is dominated by remotely forced seasonal CTWs with downwelling phases in February/March and October/November and upwelling phases in July/August and December/January^8,18^. During their propagations, CTWs induce vertical displacements of the thermocline, which influence SST, ocean biogeochemistry, and oxygen^7,19^. They also modulate the Angola Current intensity along the continental slope and shelf^20^.

**Figure 1:**
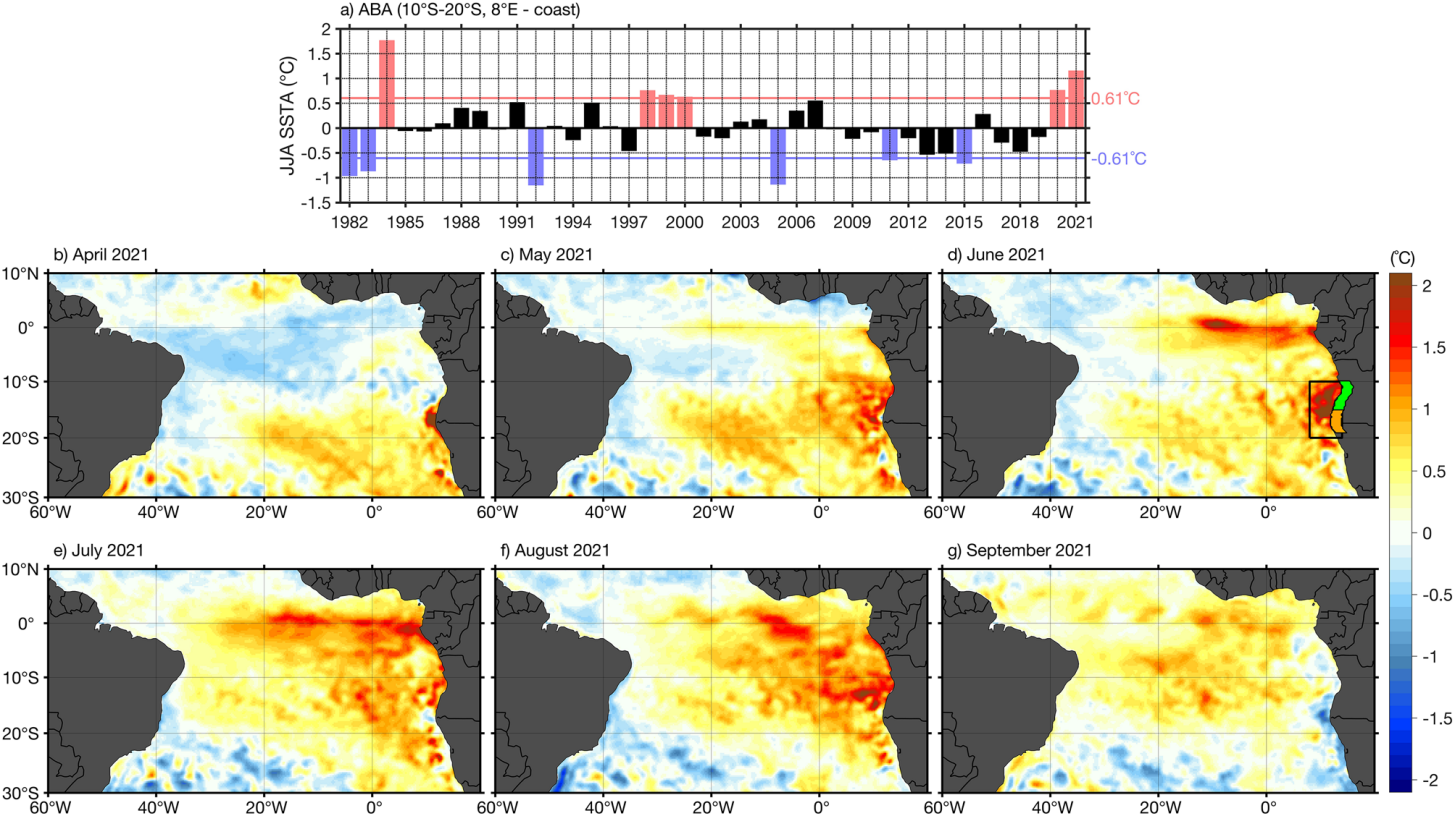
Sea surface temperature anomalies during the 2021 warm event. (**a**) Detrended June–July–August (JJA) OI-SST anomalies (SSTA) from 1982 to 2021 in the Angola-Benguela area (ABA, black box in panel d, 10°S–20°S, 8°E to the coast). The horizontal red and blue lines indicate the ± 1 standard deviation of the interannual SSTA in JJA (0.61 °C). An extreme warm (cold) event is identified when the averaged SSTA in JJA exceed ± 1 standard deviation and are represented by red (blue) bars. (**b**–**g**) Monthly detrended SSTA from April to September 2021. The 1°-width coastal band domains: The southern Angola (in green, 10°S–15°S) and the Angola-Benguela front (in orange, 15°S–19°S) are represented in panel (**d**). The anomalies are calculated relative to the period January 1982 to December 2021.

Every few years, extreme warm and cold events, so-called Benguela Niños and Niñas occur in the southeastern tropical Atlantic^21^. These extreme events can last for several months and usually peak between March and May (MAM)^12,22^ in the Angola-Benguela area (ABA, 8°E coast; 10°S–20°S, black box in Fig. 1d), an area of high interannual SST variability (Fig. 1a)^23^. They impact the regional climate^24,25^ as well as marine ecosystems and fisheries^26–28^.

Benguela Niños and Niñas can be triggered by local as well as remote forcing. Local forcing is linked to changes in the local alongshore winds, associated with the strength and position of the South Atlantic Anticyclone^29,30^. Additionally, other local processes such as freshwater input (from precipitation and Congo River discharge) and/or heat fluxes are also important as it was the case for the 1995 Benguela Niño^31^ and 2016 warm event^32^. Remote forcing is associated with CTW propagations. Moreover, modelling studies have shown that negative atmospheric feedback mechanisms taking place in the tropical Atlantic between local and remote forcing can modulate the coastal warming^33,34^. In fact, the equatorial warming triggers an anomalous converging atmospheric circulation which leads to equatorward upwelling-favorable coastal winds north of 16°S acting as a negative feedback for the coastal warming^33,34^. Furthermore, the coastal warming deepens and destabilizes the coastal marine atmospheric boundary layer. Under these conditions, equatorward surface winds intensify due to the enhanced downward mixing of momentum, favoring upwelling and evaporation and thus providing a negative feedback on local SST^34^. In return, the coastal warming also drives a positive local atmospheric response that can generate an equatorial warming^34^. Additionally, it was shown that from November to January, Pacific La Niña events can induce both remote and local forcing that contributes to the development of Benguela Niños from February to April in the northern Benguela area (17°S–25°S along the southwestern coast of Africa). The remote forcing consists of reduced zonal winds along the equator near Brazil, a key region for the formation of downwelling EKWs, whereas local forcing is linked to a weakening of the South Atlantic Anticyclone upwelling-favorable winds^35^.

During the peak season of the Benguela Niños (MAM), a study based on observations and reanalysis products indicated that interannual SST variability in the ABA has weakened by 31% during the period 2000 to 2017 relative to 1982–1999^23^. However, since 2018, the ABA has experienced a resurgence of interannual SST variability with the occurrence of two exceptionally strong coastal warm events in 2019^16,36^ and 2021^16,34,37^. This has motivated our research interests towards the interannual SST variability since 2018. In contrast to classical Benguela Niños (1995, 2001, 2010/2011), these events are atypical since they do not peak in MAM. The 2019 Benguela Niño occurred between October 2019 and January 2020 and was forced by a combination of local and remote forcing with local forcing leading the remote forcing by about one month^36^. Also, the 2021 extreme warm event occurred outside of the typical season. It peaked during or shortly before the main upwelling season in the TAUS (JAS)^34^.

Here, based on recent studies^34,37^ focussing on the physical processes associated with the 2021 extreme warm event in the ABA, we analyze the forcing mechanisms that have led to an unprecedented decrease of biological productivity in the tropical Angolan upwelling system during the main upwelling season.

## Results

### Origin, development and demise of the 2021 extreme warm event

An extreme warm event occurred in the TAUS between April and August 2021 (Fig. 1), thus covering part of the main upwelling season (JAS)^34^. For the season of June–July–August (JJA), this warm event is the second warmest ever recorded after the 1984 Benguela Niño (Fig. 1a) in the satellite era. Overall, six extreme warm (1984, 1998, 1999, 2000, 2020, 2021) and six extreme cold (1982, 1983, 1992, 2005, 2011, 2015) events have been identified in JJA. Most of those events have already been discussed in the literature^12,14,17,34^, except for the extreme warm event in 2020 and the 2015 cold event. During the onset of the 2021 event (in April, Fig. 1b), positive SST anomalies (SSTA) are observed south of 15°S with a maximum (> 1.5 °C) along the southwestern African coast between 15°S and 18°S. In May 2021, positive SSTA prevail in the whole southeastern tropical Atlantic with high SSTA spreading offshore (Fig. 1c). June 2021 marks the peak of the event with high SSTA exceeding 2 °C in the ABA (Fig. 1d). During that month, anomalous warming is observed almost in the whole southeastern tropical Atlantic with maximum SSTA (> 2 °C) located north of ~ 18°S. Simultaneously, in the equatorial Atlantic, particularly high SSTA are observed in the Atlantic Niño 3 region (20°W–0°E; 3°S–3°N), which indicates the occurrence of an Atlantic Niño^37,38^. In July 2021, positive SSTA persist in the southeastern tropical Atlantic, but are stronger in the eastern equatorial Atlantic (Fig. 1e) marking the peak of the Atlantic Niño. High coastal SSTA (> 1 °C) that spread offshore are observed north of 15°S in August 2021 (Fig. 1f). The demise of the warm event occurs in September 2021 with the appearance of negative SST anomalies (< − 0.5 °C) along the southwestern coast of Africa mostly south of 15°S (Fig. 1g).

### Contribution of the local forcing

**Figure 2:**
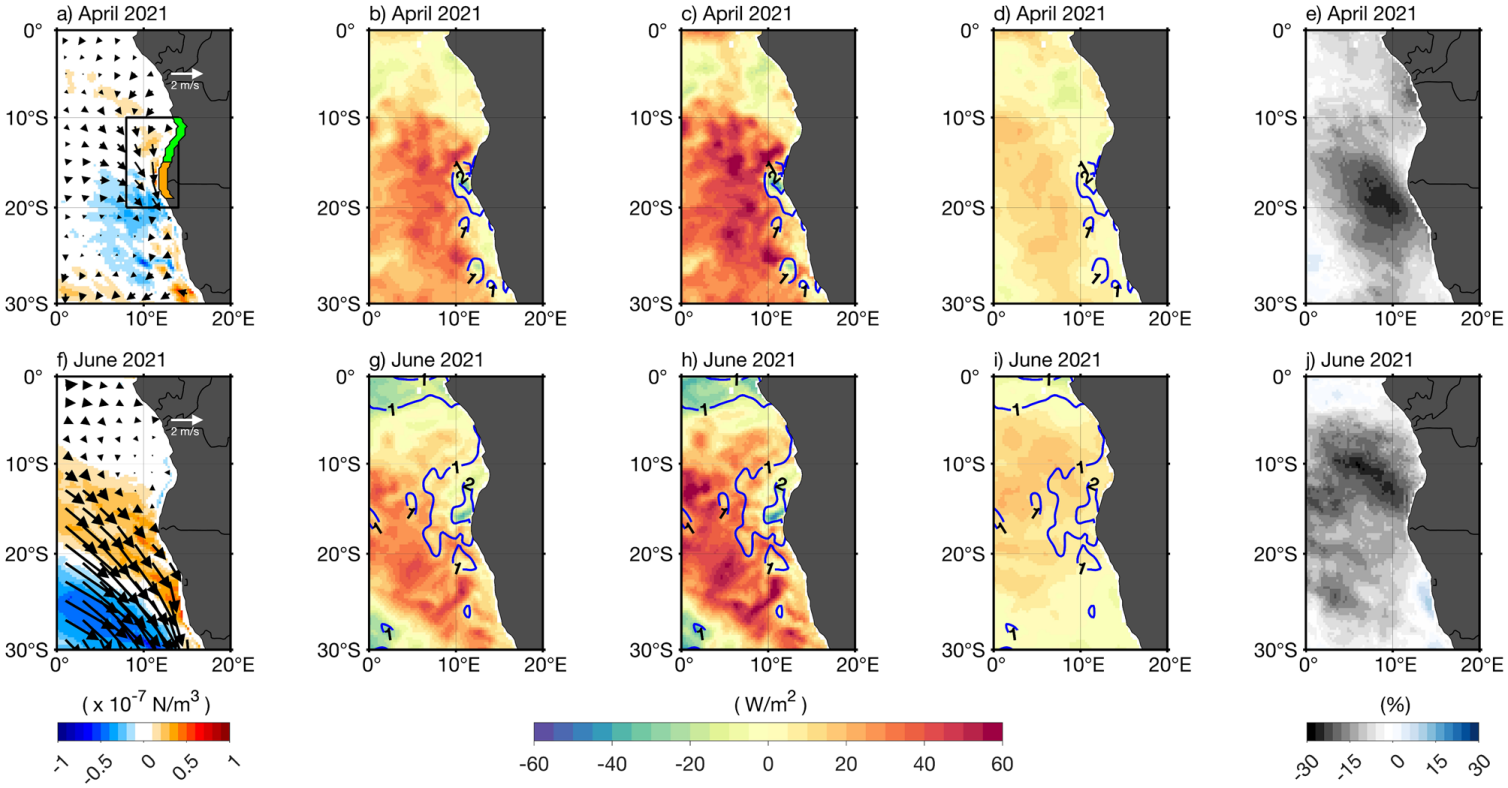
Local forcing of the 2021 warm event. Monthly detrended anomalies of: (**a**,**f**) ERA5 surface winds (arrows) and derived wind stress curl (shading), (**b**,**g**) ERA5 surface latent heat flux (shading, positive values indicate reduced latent heat loss from the ocean to the atmosphere) and SSTA (blue contours every 1 °C from OI-SST); (**c**,**h**) same as (**b**,**g**) but for the net surface heat flux anomalies (shading) and SSTA (blue contours); (**d**,**i**) same as (**b**,**g**) but for the surface shortwave radiation anomalies (shading) and SSTA (blue contours); (**e**,**j**) same as (**b**,**g**) but for the low-level cloud cover. (**a**–**e**) are for April 2021 and (**f**–**j**) for June 2021. The same coastal domains as shown in Fig. 1d are also represented here in panel (**a**): ABA (black box, 10°S–20°S, 8°E to the coast) and the 1°-width coastal band domains: the southern Angola (in green, 10°S–15°S) and the Angola-Benguela front (in orange, 15°S–19°S). The anomalies are calculated relative to the period January 1982 to December 2021.

In April 2021, a local weakening of the southeasterly winds associated with a positive WSC anomaly is observed north of 20°S (Fig. 2a). Note that a positive local near-coastal WSC anomaly indicates a weakening of the mean near-coastal cyclonic WSC, resulting in weakened Ekman suction, i.e., downwelling anomalies and reduction of the local upwelling. The reduced local upwelling, particularly around the Angola-Benguela front region (15°S–19°S, in orange, Fig. 2a) contributed to the onset of the warming in April 2021 by favoring the appearance of local positive SST anomalies (Figs. 1b, 2b).

Conversely, negative anomalous surface latent heat flux (between − 25 and −15 W/m^2^, Fig. 2b) collocating with maximum SST anomalies around the Angola-Benguela front (~ 17°S, Figs. 1b, 2b) damped the local warming in April 2021. According to Eq. (2) (see “Methods”) and Fig. S1, anomalies of the specific humidity difference between the air at 10 m and sea surface, ${q}_{a}-{q}_{o}$ (< − 2 g/kg, Fig. S1b) are the major contributor to the negative surface latent heat flux anomalies since anomalies of the wind speed are weak (between − 2 and 0 m/s, Fig. S1a) at around 17°S. The anomalies of ${q}_{a}-{q}_{o}$ are dominated by anomalies of ${q}_{o}$ (> 3 g/kg, Fig. S1d) exceeding those of ${q}_{a}$ (~ 2.5 g/kg, Fig. S1c). In fact, positive SSTA will lead to positive ${q}_{o}$ anomalies which then generate higher evaporation and negative surface latent heat flux anomalies. Reduced surface latent heat flux from the ocean to the atmosphere is mostly observed offshore. From April to May 2021, it has contributed to enhance the offshore negative SST anomaly north of 15°S and the positive anomaly south of 15°S (Fig. 1b,c). Net surface heat flux is dominated by the surface latent heat flux and thus has a similar spatial pattern but the enhanced offshore positive anomalies indicate that other terms contribute to the offshore anomalies (Fig. 2b,c). Concomitantly, positive surface shortwave radiation anomalies (< 20 W/m^2^; Fig. 2d) are observed offshore and consistent with less local low-level cloud cover (< − 15%; Fig. 2e). They have contributed to increase the offshore positive net surface heat flux anomalies in April 2021 (Fig. 2c).

**Figure 3:**
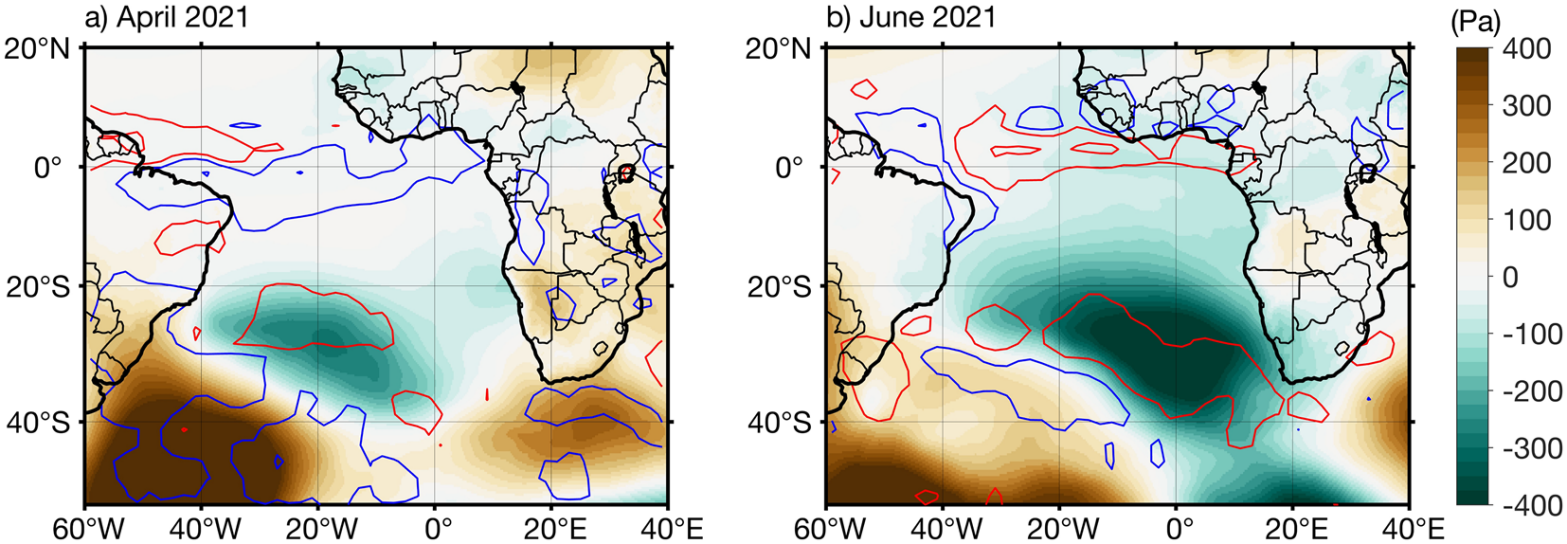
Sea level pressure and precipitation anomalies during the onset and peak of the 2021 warm event. Detrended anomalies of ERA5 monthly sea level pressure (SLP, shading) and GPCP total precipitation with red (blue) contours indicating positive (negative) precipitation anomalies (mm/day) in (**a**) April and (**b**) June 2021. Contour lines are plotted for ± 1 and ± 5 mm/day. The anomalies are calculated relative to the period January 1982 to December 2021.

Figure 3a also shows that off Namibia (south of 15°S) in April 2021, the local weakening of the southeasterly winds (Fig. 2a) occurred concomitantly with a weak seaward zonal pressure gradient associated with low (high) pressure anomaly over the ocean (on land). Indeed, negative precipitation anomalies (~ − 1 mm/day) are observed on land linked to high surface pressure anomalies, but also along the equator with the intertropical convergence zone shifted to the North (Fig. 3a).

In June 2021, the high-pressure system linked to the South Atlantic Anticyclone was significantly reduced with SLP anomalies exceeding − 400 Pa (Fig. 3b) and associated with a strong weakening of the southeasterly winds (Figs. 2f and S2c). The weakening of the winds (cf*.* Fig. S2c) is consistent with positive WSC anomalies (> 0.3 × 10^–7^ N/m^3^) observed from the southwestern African coast to a few degrees offshore south of 10°S (Fig. 2f). It resulted in weakened coastal upwelling and a reduction of latent heat loss (> 30 W/m^2^, Fig. 2g) from the ocean to the atmosphere enhancing the warming in the region south of 17°S.

Figure S1f shows that anomalies of ${q}_{a}-{q}_{o}$ drove the negative surface latent heat flux anomalies located along the southwestern African coast between 10°S and 17°S which damped the positive SSTA from June to July 2021 (Fig. 1d,e). Conversely, wind speed anomalies drove the positive surface latent heat flux anomalies along the southwestern African coast south of 17°S and offshore (Fig. S1e) that enhanced the positive SSTA from June to July (Fig. 1d,e). As for April 2021, net surface heat flux anomalies (Fig. 2h) depict quite similar patterns as surface latent heat flux anomalies.

Between 5°S and around 20°S along the southwestern African coast, positive anomalies of shortwave radiation (> 5 W/m^2^; Fig. 2i) associated with a reduction in the low-level cloud cover (< − 15%; Fig. 2j) might have contributed to the warming. The peak of the event is not linked to local precipitation anomalies (Fig. 3b). Instead, high precipitation anomalies (> 3 mm/day) are observed in the South Atlantic associated with negative anomalous SLP (Fig. 3b) and also north of the equator linked to the onset of the Atlantic Niño (Fig. 1d). Therefore, like the recent 2019 Benguela Niño, the 2021 extreme warm event was not linked to local precipitation anomalies.

### Contribution of the remote forcing

**Figure 4:**
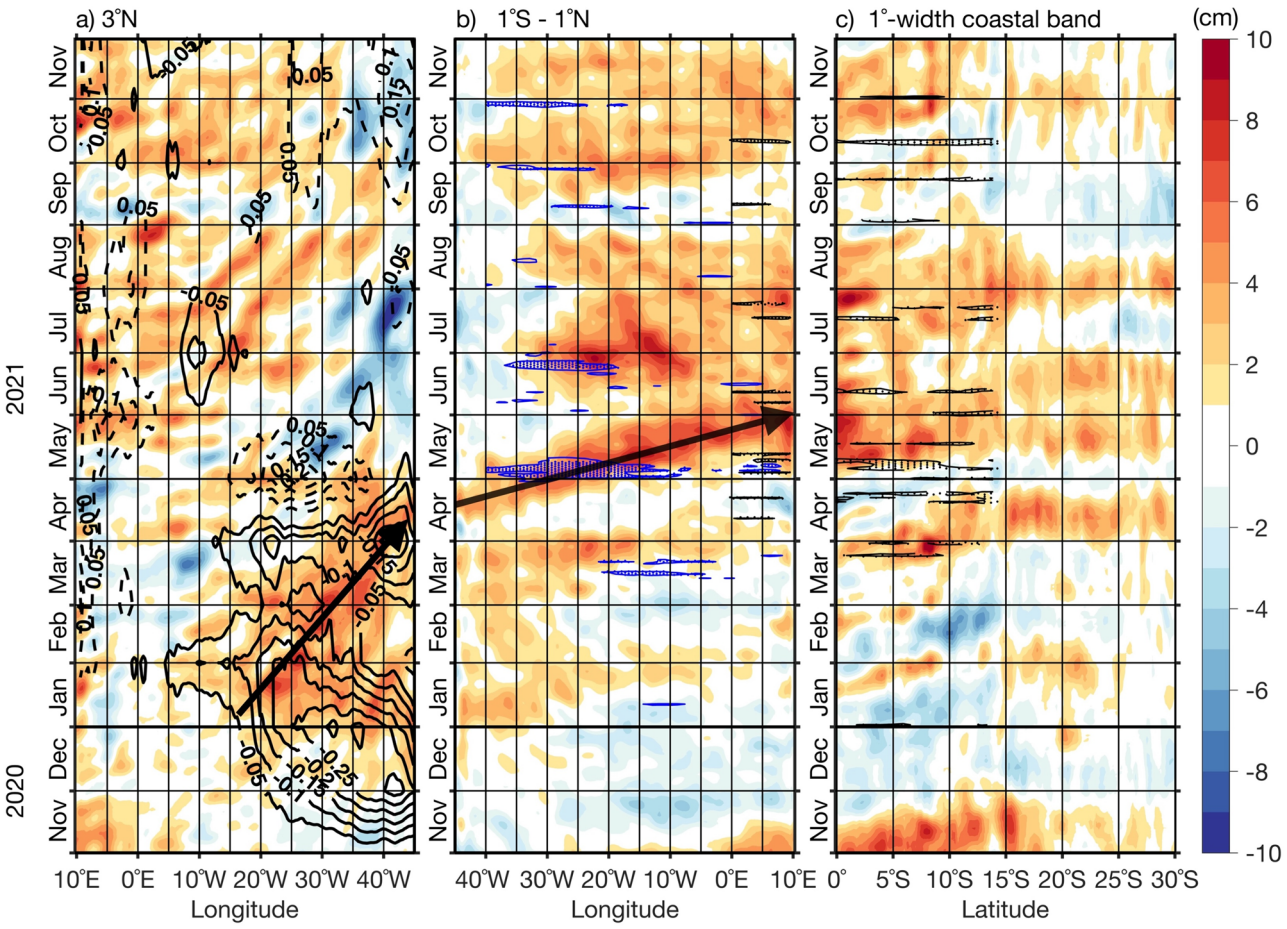
Equatorial and coastal trapped wave propagations during the 2021 warm event. (**a**) Hovmöller diagram of detrended SLA (shading) at 3°N and WSC anomalies (black contours; 1e−7 N/m^3^) averaged over 0°N–3°N from 45°W to the African coastline. (**b**) Same as (**a**) but for SLA (shading) averaged between 1°S and 1°N along the equator and ERA5 surface zonal wind anomalies (blue dotted contours highlighting anomalies > 3 m/s) and meridional wind anomalies (black dotted contours highlighting anomalies < − 2 m/s) along the equator and averaged over 3°S–3°N. (**c**) Same as (**a**) with SLA along the southwestern African coast and ERA5 surface meridional wind anomalies (black dotted contours highlighting anomalies < − 2 m/s) averaged within 1°-width coastal band from 0°S to 30°S. Data are shown from November 2020 (bottom) to November 2021 (top). Note that the x-axis in (**a**) has been reversed to help visualizing wave reflection at the western boundary. The anomalies are calculated relative to periods January 1993 to December 2021 (SLA) and January 1982 to December 2021 (zonal wind and WSC). The black arrows in panels (**a**) and (**b**) highlight the propagation of positive SLA signals.

WSC fluctuations in the western off-equatorial Atlantic induce sea level anomaly (SLA) variability via forcing of Rossby waves^39^. At 3°N (Fig. 4a), west of 15°W, positive sea level anomalies (SLAs) are observed between January and April 2021. The most prominent positive SLA developed at ~ 16°W at the beginning of January, intensified as it propagated westward, and reached the western boundary on ~ 10th of April 2021. This corresponds to a propagation speed (see methods) of ~ 0.38 m/s^37^, which is close to the phase speed of the first meridional mode Rossby wave of the second baroclinic mode^40^. This downwelling Rossby wave was triggered and enhanced by the Ekman convergence from persistent and negative WSC anomalies (solid contours in Fig. 4a; Fig. S3) between January and March 2021^37^.

At the western boundary, the slowly westward propagating positive SLA signal reflected into a positive SLA signal propagating eastward along the equator starting from mid-April and reaching the eastern boundary at the end of May 2021 (Fig. 4b). The estimated speed of the positive equatorial SLA signal propagation is ~ 1.8 m/s (see “Methods”), which is close to that of an EKW of second baroclinic mode^34,37,40^. During its propagation along the equator, the downwelling EKW of the second baroclinic mode will deepen the thermocline and induce positive subsurface temperature anomalies^34,37^.

Further, additional forcing of downwelling EKWs stems from positive anomalous equatorial zonal winds denoted by dotted blue contours (> 3 m/s) in the western and central equatorial Atlantic in the first half of May 2021 (Figs. 4b, S2a)^37^. This explains the enhanced amplitude of the SLA signal (> 7 cm).

Surface wind fluctuations might have caused additional local warming at the eastern boundary (Fig. 1). There, Fig. 4b shows a positive SLA signal (> 6 cm) already in early May 2021 before the passage of the downwelling EKW. This may be associated with prevailing relaxed cross-equatorial southerly winds (< − 2 m/s, black dotted contours in Fig. 4b) east of 0°E in May. They may have induced the eastern equatorial warming (see Fig. 1c) and positive SLAs in agreement with *Philander and Pacanowski*^41^. This was also observed during the 2019 Benguela Niño event^36^. In addition to suppressed cross-equatorial southerly winds, downwelling EKWs forced by westerly wind anomalies (> 3 m/s, blue dotted contours in Figs. 4b; Fig. S2a) observed east of 0°E might have also generated positive SLAs in May 2021.

Along the southwestern African coast, downwelling CTW propagations associated with SLAs higher than 5 cm are observed from the end of March to April and from May to August 2021 with a connection to the equatorial Atlantic (Fig. 4b,c). Their signatures can be tracked down to ~ 30°S. They have generated positive SST anomalies during the event. Between 0°S and 10°S, weakened coastal southerly winds prevailed with anomalies lower than − 2 m/s (black dotted contours in Fig. 4c; Fig. S2c) observed at the end of March and from end of April to early May 2021. They provided additional forcing of downwelling CTWs especially at the end of March since enhanced amplitude of SLAs (> 4 cm) is observed along the southwestern African coast. Thus, remote and local forcing have contributed to set up the extreme warm event in April 2021 north of 20°S. Our result agrees with *Illig and Bachèlery*^34^ who also observed a downwelling CTW propagation between March and April 2021.

### Role of the advection during the 2021 extreme event

**Figure 5:**
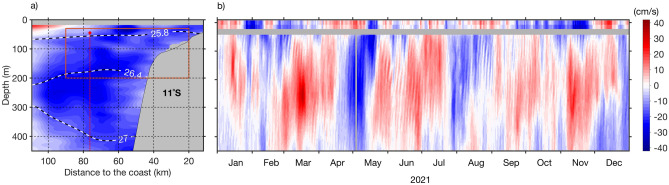
Poleward flow along the Angolan coast. (**a**) Shipboard alongshore velocity section at 11°S taken on May 3, 2021 during RV Sonne cruise SO283. Thick dashed black and white contours depict isopycnals ${\sigma }_{\theta }$ = 25.8, 26.4 and 27 kg/m^3^. The dashed red line represents the mooring position. The red box shows the area used to calculate the Angola-Current transport (20–90 km and 20–200 m-depth). (**b**) Time series of daily alongshore velocities from Globcurrent (at the surface and at 15-m depth) and from a moored ADCP (below 45-m depth) at 13°00′E; 10°50′S for 2021. The grey shaded areas in (**a**,**b**) highlight missing data.

A shipboard alongshore velocity section from early May 2021 shows a strong vertically coherent poleward flow (< − 25 cm/s) in the upper 450 m at 11°S within 100 km of the coast (Fig. 5a). This indicates a strong Angola Current associated with the passage of a low-mode downwelling CTW in April 2021 (Fig. 4c). This low-vertical-mode structure in the alongshore velocity section is also observed in moored alongshore velocities in early May 2021 (Fig. 5b). Note that the position of the 11°S mooring is represented by the vertical dashed line in Fig. 5a. A poleward transport of the Angola Current of 2.1 Sv (1 Sv = 10^6^ m^3^/s) has been derived by integrating the shipboard alongshore velocity over the red box (Fig. 5a), as done previously by Kopte et al.^42^. This poleward transport is substantially higher than the mean poleward transport of the Angola Current of 0.32 Sv as estimated by Kopte et al*.*^42^. The strong poleward flow of the Angola Current in May 2021 could have contributed to the development of the warm event through advection of subsurface warm equatorial waters downstream. Moreover, alongshore current velocities from Globcurrent at 11°S (Fig. 5b) display a continuously poleward current (~ 15 cm/s) around mid-May in the upper 15 m. Warm equatorial surface waters might have been advected poleward by the surface currents and generate positive SSTA downstream. The bending of the isopycnals 26.4 and 27 kg/m^3^ shown in Fig. 5a, also indicates the presence of higher-mode structures^10^.

**Figure 6:**
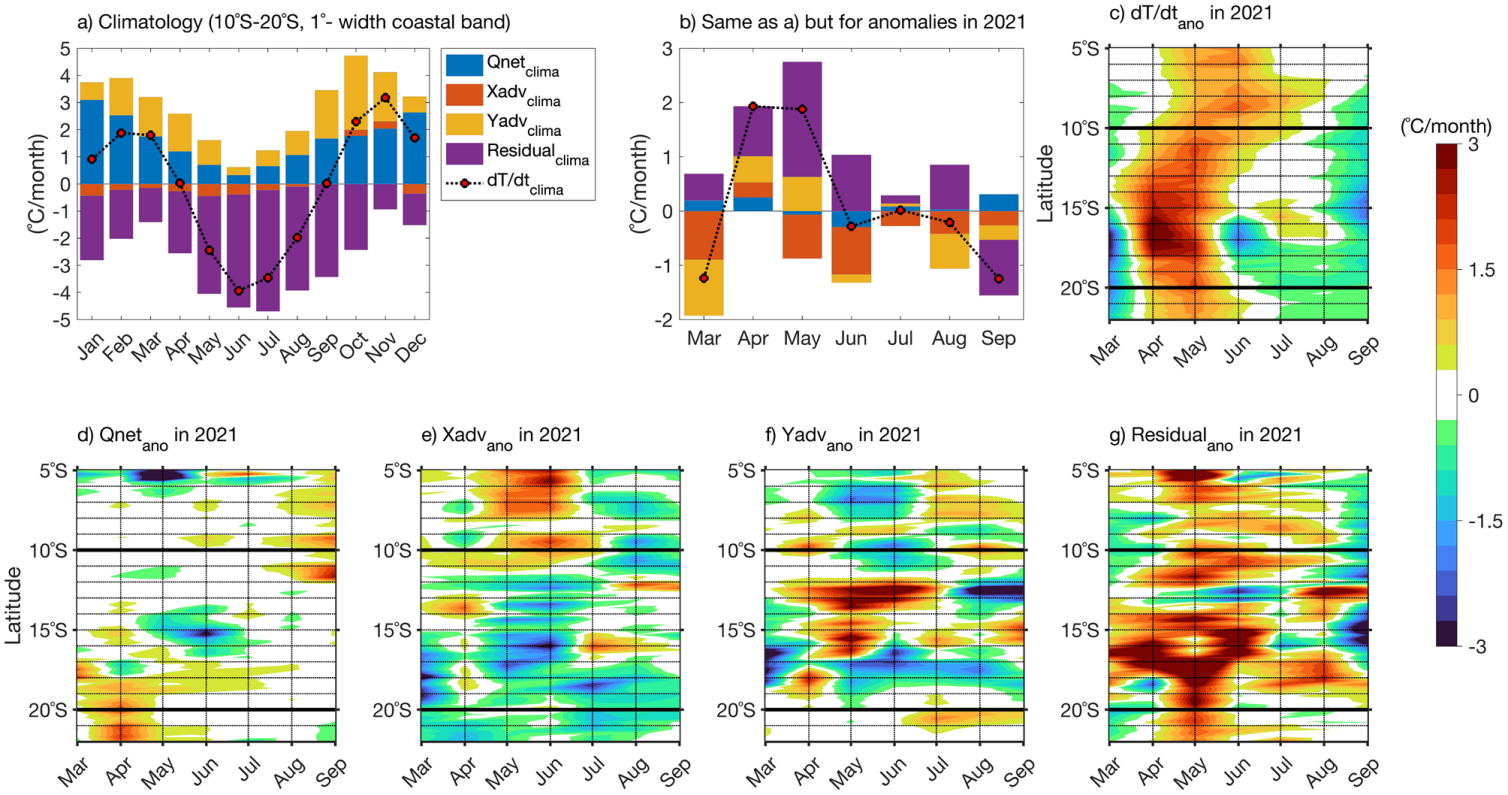
(**a**) Contribution of the heat budget terms to the warming. (**a**) Climatological contribution of each term of the mixed layer heat budget from 1982 to 2021 and averaged between 10°S and 20°S within the 1°-width coastal band. (**b**) Same as (**a**) but for detrended monthly anomalies during March to September 2021. (**c**) Hovmöller diagram of detrended monthly anomalies of temperature tendency averaged between the surface and the mixed layer depth within 1°-wideband along the southwestern African coast from 5°S to 22°S. (**d**–**g**) Same as (**c**) but for surface net heat fluxes, zonal and meridional heat advection and residual, respectively. (**b**–**g**) Data are shown from March (left) to September 2021 (right). Anomalies are calculated relative to the period January 1982 to December 2021. The black horizontal lines in panels (**c**–**g**) represent the latitudinal boundaries on the ABA.

To quantify the contribution of the meridional advection and the other terms during the extreme warm event, an analysis of the mixed layer heat budget is performed utilizing ORAS5 reanalysis in the southeastern tropical Atlantic (Fig. 6). Climatologically, the analysis reveals that from 10°S to 20°S within the 1°-width coastal band, the surface warming during October to March is mostly explained by net surface heat fluxes with an additional contribution due to meridional advection (Fig. 6a) and a smaller contribution only during October to November due to zonal advection. These results agree with the ones of Körner et al.^43^.

However, the residual term strongly cools down the mixed layer temperature throughout the year and is very large. According to Körner et al.^43^, in this region, the heat flux due to turbulent mixing across the base of the mixed layer which is included in the residual term, is an important cooling term and could explain the large values in the residual. Similar to Fig. 6a, anomalies of each term of the mixed layer heat budget between March and September 2021 are shown in Fig. 6b. Overall, an anomalous residual term prevails as the main contributor to warming up the mixed layer during the 2021 extreme event. These large residual anomalies might also come from the warming induced by a reduced entrainment of cold subsurface waters into the surface mixed layer due to the depressed thermocline during propagations of downwelling CTWs along the coast.

During April 2021, all terms contribute to the anomalous warming of the mixed layer (1.93 °C/month, Fig. 6b) with 48%, 25%, 14% and 13% explained by anomalies of the residual, meridional advection, zonal advection and net surface heat flux, respectively. In addition to the huge warming caused by the anomalous residual term (see Fig. 6g), warming due to anomalous zonal advection in April 2021 (Fig. 6e) is consistent with the weakening of local alongshore winds north of 20°S (Fig. 2a). This is linked to an anomalous onshore transport of warm surface waters consistent with the reduction of the local upwelling that could have contributed to the positive anomaly. In April 2021, there is a cooling due to anomalous net surface heat fluxes (between − 1 and 0 °C/month, Fig. 6d, cf. Fig. 2b,c) between 17°S and 18°S where the maximum SSTA is located (Figs. 1b, 2b). However, when averaging between 10°S and 20°S, this cooling is overcome by the warming north of 16°S and south of 18°S, resulting in a net warming in the coastal band during April (Fig. 6b).

In May 2021, anomalies of the residual and meridional advection contribute to 77% and 23% of the anomalous warming terms (2.75 °C/month, Fig. 6b), respectively. In fact, only anomalous meridional advection has contributed to the warming (> 2 °C/month) beside the important anomalous residual term and is strong between 12°S and 17°S (Fig. 6f). This is consistent with observations at 11°S showing an anomalous poleward flow which could have transported warm equatorial waters poleward, thereby contributing to the warming in the ABA.

### Impact on the biological productivity

**Figure 7:**
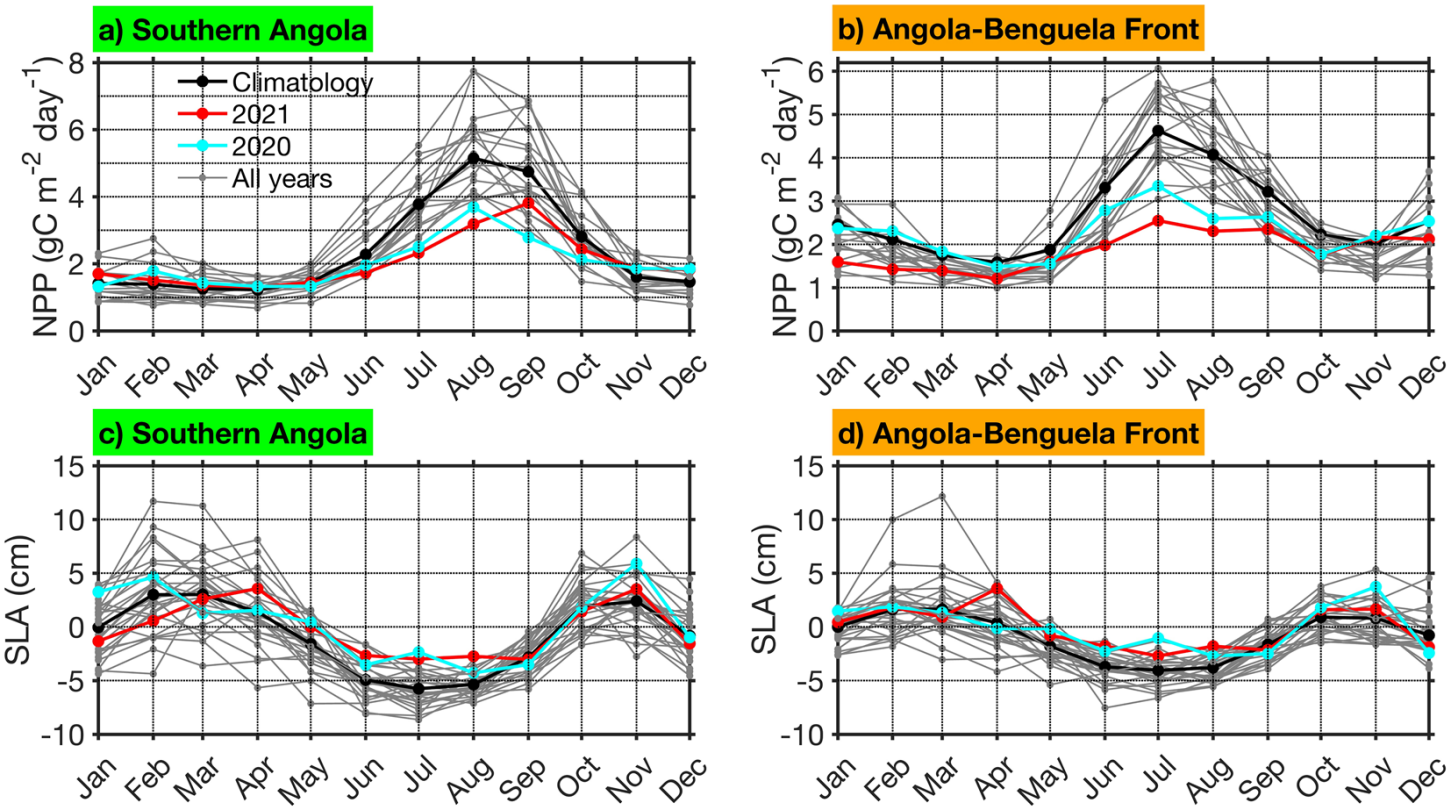
Impact of extreme warm events on net primary production. NPP (gC m^−2^ day^−1^) for all years from 2002 to 2019 (grey lines) averaged in the domains (**a**) southern Angola (10°S–15°S, 1°-width coastal band, green in Fig. 1d and (**b**) Angola-Benguela front (15°S–19°S, 1°-width coastal band, orange in Fig. 1d, plotted as 12-month segments with 2020 (cyan line), 2021 (red line) and the climatology (black line) as noted in the legend in panel a), respectively. (**c**,**d**) Same as (**a**,**b**) but for SLA (cm).

The tropical Angolan upwelling system is a highly productive marine ecosystem. It is associated with high net primary production (NPP) during its main productive season (JAS) in southern Angola (Fig. 7a). The peak of the NPP in JAS is remotely forced from the equatorial Atlantic by the major upwelling CTW phase in JJA^18^ that is marked by negative SLA (Fig. 7c, black line). This upwelling CTW signature is still observed in the Angola-Benguela front domain (Fig. 7d) and drives the local high NPP in July–August (Fig. 7b) in combination with alongshore upwelling-favorable winds^4,12^.

During the 2021 extreme warm event, in the coastal domains off southern Angola and the Angola-Benguela front, unprecedented low NPP records were reported (red line) with respect to the climatology (black line) between 2002 and 2021 (Fig. 7a,b). For example, during the extreme warm event, low values of NPP were observed in August (3.19 gC m^−2^ day^−1^, red line, Fig. 7a) off southern Angola and in July (2.55 gC m^−2^ day^−1^, red line, Fig. 7b) in the Angola-Benguela front domain and collocated with positive SSTA (see Fig. 1). These values are lower than the climatology of the corresponding month in each domain, i.e. in August (5.15 gC m^−2^ day^−1^, black line; Fig. 7a) off southern Angola and in July (4.63 gC m^−2^ day^−1^, black line; Fig. 7b) in the Angola-Benguela front domain. This is consistent with Fig. S4 which illustrates a strong reduction in NPP along the southwestern African coast during the extreme warm event in July and August 2021 north of 20°S. We further notice that the 2020 extreme warm event also had an impact on the NPP in the two coastal domains (Fig. 7a,b), although weaker than that of the 2021 extreme warm event.

Off southern Angola, in JJA 2021, positive coastal SLA of 2.55 cm was observed compared to the climatology (Fig. 7c) and associated with a weak upwelling CTW (downwelling SLA signal). The downwelling SLA signal in 2021 was one of the strongest during JJA between 2002 and 2021 and has fairly comparable magnitude to the one in 2020. Consequently, this poleward propagation of positive SLAs off southern Angola reduced the wave-driven thermocline upwelling near the coast during the main upwelling season. A similar mechanism was found in a previous modelling study^13^. The reduced local upwelling resulted in a reduced downward heat flux and surface warming and at the same time in a reduced upward flux of nutrients into the euphotic zone. Hence, the biological productivity off southern Angola was reduced during the warm event in 2021. The downwelling SLA signal reached the Angola-Benguela front domain, where similar impacts were observed in NPP. In addition to the downwelling SLA signal, in the Angola-Benguela front domain, the strong reduction of the southeasterly winds (Figs. 2f, S2c) consistent with the positive, i.e., downwelling-favorable WSC anomalies observed in June 2021 have resulted in the reduction of the local upwelling as well as biological productivity.

## Discussion and conclusions

In this study, we used observations and reanalysis data to describe the phenology and the forcing mechanisms of the 2021 extreme warm event as well as its impact on the biological productivity in the tropical Angolan upwelling system. The warm event took place between April and August 2021 (Fig. 1), thus covering part of the main upwelling season off Angola. It was atypical since it peaked in June with SSTA exceeding 2 °C in the ABA (Fig. 1d) while canonical Benguela warm/cold events usually peak in March–April–May^17^. SSTA averaged over the ABA for JJA revealed that the 2021 extreme warm event was the second strongest austral winter event recorded in the satellite era after the 1984 Benguela Niño event (Fig. 1a). This is also confirmed with HadISST (Fig. S5) for the period 1982–2021, despite the difference in the number of identified extreme events compared to OI-SST (Fig. 1a).

As reported in previous studies, canonical Benguela Niños were dominantly forced by remote forcing^13,14,44,45^. However, the two most recent extreme warm events that occurred in the ABA in 2019 and 2021 were forced by a combination of local and remote forcing. We show here that the onset of the warming in April 2021 was initiated by: (1) a weakening of the local southeasterly winds^34^ associated with positive, i.e. downwelling-favorable WSC anomalies (Fig. 2a) and (2) remotely forced downwelling CTW, both resulting in a reduction of local upwelling. Similarly, both forcings jointly acted during the peak of the extreme warm event as also shown by *Illig and Bachèlery*^34^. A pronounced weakening of the southeasterly winds (Fig. 2f) associated with a considerably reduced high-pressure system in the South Atlantic (Fig. 3b) favored enhanced warming in the ABA. This result is consistent with the modelling study of *Illig and Bachèlery*^34^ which showed that the 2021 Benguela Niño was triggered by anomalous atmospheric fluxes at their model’s southern boundary (32°S), related to a significant and persistent weakening of the South Atlantic Anticyclone. The remote forcing consisted of a slow downwelling Rossby wave that propagated between January and April 2021 (Fig. 4a) forced by persistent negative WSC anomalies at 3°N in the western tropical Atlantic between January and March 2021 (Figs. 4a and S3). This Rossby wave was reflected at the coast of South America as a downwelling EKW in early April 2021. Upon arrival at the eastern boundary, the eastward propagating downwelling EKW triggered a downwelling CTW that propagated along the southwestern African coast, imprinting the coastal SLA up to 30°S (Fig. 4c). Moreover, the downwelling EKW signal was amplified by a strong weakening of equatorial zonal winds in the first half of May (blue dotted contours in Fig. 4b). This result is in agreement with previous studies^34,37^ which also indicated that between the 1st and 7th of May 2021, the pronounced reduction of equatorial zonal winds (blue dotted contours in Fig. 4b) called westerly wind bursts in the study by Lee et al.^37^, was driven by the Madden–Julian oscillation and involved in the forcing of the 2021 Atlantic Niño^37^.

Northerly wind anomalies exceeding − 2 m/s (black dotted contours in Fig. 4c) at the end of March and from end of April to early May 2021 between 0°N and 10°S provided additional forcing of poleward propagating downwelling CTWs. A previous study^17^ already emphasized the importance of northerly wind anomalies north of 10°S in generating additional coastal SLA variability along the southwestern African coast through triggering downwelling CTWs. These CTWs can play a significant role by enhancing positive SST anomalies during warm events as it was the case for the 2019 Benguela Niño^36^.

Previous studies^26,36^ highlighted the impact of Benguela Niños on the biological productivity in the ABA in other seasons outside the main upwelling season (JAS). This study reveals for the first time that the 2021 extreme warm event, ranked the second extreme warm event in the satellite era in JJA (Figs. 1a, S5), has the strongest impact on the biological productivity in the southern Angola and Angola-Benguela front domains between 2002 and 2021 (Fig. 7a,b). It is unfortunate that this relationship cannot be evaluated for the other extreme warm events identified before 2002 (Fig. 1a) due to the lack of NPP data. The anomalously weak NPP was caused by reduced upwelling associated with weaker alongshore winds and positive WSC anomalies in June (Fig. 2f) as well as the propagation of weak upwelling CTW (visible as a downwelling SLA signal) in JJA (Figs. 4c, 7c,d). We suggest that the combination of these two forcing mechanisms during the main upwelling season can explain this unprecedented decrease in the NPP in the tropical Angolan upwelling system.

Finally, the 2021 extreme warm event peaked one month before the equatorial Atlantic Niño reached its maturity in July 2021 (Fig. 1d,e). Previous and recent studies using satellite, reanalysis data and numerical experiments evidenced that locally forced warming over the ABA is likely to generate westerly wind anomalies along the equatorial Atlantic one to two months later which will be linked to a warming in the equatorial Atlantic^33,46^. That was the case for the 2019 Atlantic and Benguela Niños^36,38^ and as shown in the present study for the 2021 Atlantic Niño and ABA extreme warm event^34,38^. However, the timing of the triggering mechanisms of the warm event in the ABA is crucial for the development of the Atlantic Niño^33,34^, and as already suggested in Lübbecke et al.^22^ and Nnamchi et al.^47^, these two extreme warm events could be seen as one.

## Data and methods

### Data

We use monthly mean SSTA from the daily Optimum Interpolation SST version 2 (OI-SST)^48^ available at 0.25° horizontal resolution. These are obtained from daily merged remotely sensed and in situ data. The period used here spans from January 1982 to December 2021.

The European Centre for Medium-Range Weather Forecasts reanalysis 5 (ERA5)^49^ is used to analyze atmospheric variables, for instance surface pressure, surface winds, low-level cloud cover, specific humidity of the air, air-sea heat fluxes and sea level pressure (SLP) as in Imbol Koungue et al.^36^. The data are available at 0.25° horizontal resolution and we use monthly means for the period January 1982 to December 2021.

The impact of the extreme warm event on the local marine ecosystem is assessed by analyzing the local net primary production (NPP) from the Eppley vertically generalized production model^50^. It is based on MODIS chlorophyll, SST data, photosynthetically active radiation and estimates of the euphotic zone depth. For this study, monthly NPP data for the time period July 2002 to December 2021 are used. The dataset has a horizontal resolution of 1/6° but is interpolated onto a 0.25° × 0.25°Spatial grid to match the SST anomalies^36^.

Monthly precipitation data is taken from the Global Precipitation Climatology Project version 2.3 (GPCP)^51^. It is a blend of satellite and station data available at 2.5° horizontal resolution. We use data from January 1982 to December 2021.

We also use monthly mean SST data from the Hadley Centre Sea Ice SST data set Version 1.1 (HadISST)^52^ to assess the robustness of our results, especially for the JJA ABA-averaged SSTA. The data have a 1° horizontal resolution and are available from January 1870 to December 2022.

Off Angola at 13°00′ E, 10°50′ S around 77 km away from the coast, current velocity measurements are available from a mooring since July 2013^20,42^. The mooring is installed at the continental slope at about 1200 m water depth. On the mooring cable, at 500-m depth, an upward-looking 75-kHz Long Ranger acoustic Doppler current profiler (LR ADCP) is mounted to measure the velocity of the Angola Current up to 45 m below the sea surface with a 16-m bin size as vertical resolution^42^. Current velocities are rotated 34° anticlockwise of north to derive alongshore and cross-shore velocities (positive onshore) according to the local coastal orientation^36^. Also, a cross-shelf section with a shipboard 75-kHz ADCP performed at about 11°S during the RV Sonne cruise SO283 in May 2021 is used to display alongshore velocity in the upper 450 m.

Daily SLA from the delayed-time multi-mission (all satellites merged) product is used. SLA is distributed by the European Union Copernicus Marine Service Information. The data are available at 0.25° horizontal resolution and we use the period from January 1993 to December 2021.

A gridded product of total current velocities (Ekman + geostrophic currents) from Globcurrent at the surface and 15-m depth^53^ is also used. Current velocities are extracted from the data point 13°07.5′ E, 10°52.5′S that is closest to the mooring position to complement the moored velocity time series^36^. Similar to SLA data, these current velocities are available at a daily temporal resolution and are distributed by the European Union Copernicus Marine Service Information. They are available at 0.25° horizontal resolution from January 1993 to July 2022.

Mixed layer heat budget terms, except for the net surface heat flux contribution derived from ERA5, are calculated using the Ocean Reanalysis System 5 (ORAS5)^54^. Monthly mean ORAS5 data are available from January 1958 onwards with a horizontal resolution of 0.25° and on 75 vertical levels. ORAS5 has a 5-day assimilation cycle for observations, which include SST, subsurface temperature and salinity, and SLA. Before calculating the mixed layer heat budget terms, a comparison between ORAS5 and observations was made. First, the accuracy of ORAS5 to represent the SSTA with respect to the climatology (1982–2021) during the onset (April, Fig. S6a,c) and the peak (June, Fig. S6b,d) of the 2021 extreme warm event was evaluated against OI-SST. In general, ORAS5 compares well to OI-SST in April and June 2021 in terms of the intensity and location of the maximum SSTA. Second, we have compared the seasonal cycle of moored and total alongshore current velocities at 11°S with the one from ORAS5 derived by averaging the grid points closest to the mooring position between August 2013 and December 2021 (Fig. S6e,f). Seasonally, strong equatorward flow (> 10 cm/s) is observed at the surface from April to August and from December to January in ORAS5 (Fig. S6f) that is not captured by the alongshore velocities in the upper 15 m (Fig. S6e). ORAS5 compares well to the seasonal cycle for currents between 45 and 60 m from the moored ADCP (which provides data up to 45 m below the surface), with a primary maximum of the southward current (< − 12 cm/s) in October and a secondary poleward flow maximum between January and March. The seasonal cycle can be described as a superposition of a semiannual and annual harmonic of alongshore velocity characterizing the Angola Current^55^. Below 100 m, moored velocities portray a weak equatorward flow from January to May (< 4 cm/s) and maximum equatorward flow between October and December (> 8 cm/s), whereas poleward flow is observed between July and September (Fig. S6e). These patterns are quite well represented in ORAS5 despite having a weaker amplitude. Overall, ORAS5 reasonably represents available independent observations.

### Methods

Prior to calculating anomalies for the different parameters and the heat budget terms, the linear trend estimated over each data set period (see data section for each data time period) has been removed and anomalies are calculated with respect to the corresponding climatology. Surface net heat flux (${Q}_{net}$) is a combination of the ERA5 radiative (surface shortwave and longwave radiations, respectively ${Q}_{swr} \,{\text{and}}\, {Q}_{lwr}$) and turbulent fluxes (surface latent and sensible heat fluxes, respectively ${Q}_{lat}\, \text{and}\, {Q}_{sen}$) as follows:

1 $$ Q_{net} = Q_{swr} + Q_{lwr} + Q_{lat} + Q_{sen} $$

All surface heat fluxes are positive downward. To investigate the drivers of anomalous ${Q}_{lat}$ during the extreme warm event, we use the following bulk formula expressing ${Q}_{lat}$, in terms of the contribution from the wind speed and specific humidity difference between the air at 10 m and sea surface:

2 $${Q}_{lat}={\rho }_{a}{C}_{e}{l}_{v}({q}_{a}-{q}_{o}){U}_{10}$$

where ${\rho }_{a}$ = 1.225 kg/m^3^ is the air density; ${l}_{v}$ = 2.50*10^6^ J/kg is the latent heat of evaporation; ${C}_{e}$ is the transfer coefficient for water vapor (see^56^ for calculation); ${U}_{10}$ is the wind speed at 10 m from ERA5; ${q}_{\text{o}}$ is the saturated specific humidity at the temperature of the sea surface estimated from ERA5 SST and surface pressure (Eqs. 3 and 4); and ${q}_{a}$ is the specific humidity of air at 10 m from ERA5. The saturated specific humidity at the temperature of the sea surface has been estimated using the formula^57^:

3 $${q}_{o}= \frac{\frac{{R}_{dry}}{{R}_{vap}}{e}_{s}(T)}{P-(1-\frac{{R}_{dry}}{{R}_{vap}}){e}_{s}(T)}$$

4 $${e}_{s}\left(T\right)= {a}_{1}exp\left[{a}_{3}\left(\frac{T- {T}_{0}}{T- {a}_{4}}\right)\right]$$

where ${R}_{dry}$ = 287.0597 J/(kg K) and ${R}_{vap}$ = 461.5250 J/(kg K) are the gas constants for dry air and water vapor, respectively. P and T are the surface pressure (in Pa) and the SST (in K) from ERA5 reanalysis, respectively. T_0_ = 273.16 K and ${e}_{s}\left(T\right)$ is the saturation vapor pressure (in Pa) derived from ERA5. The parameters ${a}_{1}$, ${a}_{3}$ and ${a}_{4}$ are set to 611.21 Pa, 17.502 and 32.19 K, respectively^57^.

The positive SLA propagation speeds ${v}_{sla}$ are estimated using ${v}_{sla}= \frac{dx}{dt}$, with $dx$ being the distance along the (equatorial/off-equatorial) wave guide and $dt$ the corresponding lag. Along the equator, using Fig. 4b, $dt$ is estimated based on a lag correlation analysis between detrended SLAs and SLAs averaged between 25°W and 23°W from April, 1 to June, 15 2021. Similarly, off the equator, at 3°N using Fig. 4a, $dt$ is estimated based on a lag correlation analysis between detrended SLAs and SLAs averaged between 20°W and 15°W from December, 1 to April, 30 2021.

To understand the processes that governed the temperature variations during the 2021 extreme warm event, a mixed layer heat budget analysis is performed using the monthly ORAS5 product. The mixed layer depth (MLD) is defined as the depth at which temperatures deviate by 0.2 °C with respect to the reference depth (ref)^43,58^, here defined as the first level of the ORAS5 product (i.e., ~ 0.5 m). The mixed layer heat budget equation is as follows^43,59,60^:

5 $$\frac{\partial {\text{T}}}{\partial {\text{t}}}=-v.\nabla {\text{T}}+\frac{{q}_{net}}{\rho {c}_{p }h}+{\text{ r}}$$

where ρ is the seawater density (i.e., 1025 kg/m^3^) and ${c}_{p}$ the specific heat capacity of water (i.e., 4000 J/(kg K)). $h$ represents the MLD, and $T$ indicates the mean mixed layer temperature. $v$ represents the horizontal velocity vector vertically averaged for the MLD. From left to right each term denotes respectively: mixed layer temperature tendency; mixed layer horizontal temperature advection, net surface heat flux corrected for the ${Q}_{swr}$ that penetrates below the MLD and a residual. The ERA5 reanalysis was used to calculate the net surface heat flux term (${Q}_{net}$) as shown in Eq. (1), with ${Q}_{swr}$ replaced by the shortwave radiation absorbed by the mixed layer (${Q}_{abs}$), defined as: ${Q}_{abs}={Q}_{swr} (1-0.47{e}^{-h/15})$, taking an albedo rate of 6% where $h$ is the MLD in meters^61^. Terms not represented in Eq. (5) (e.g., turbulent heat loss, vertical temperature/velocity covariance, entrainment)^43,59–61^ are included in the residual term. The residual also contains the contribution of processes unresolved by the spatial and temporal scales of the reanalysis product. All terms from Eq. 5 are obtained for the period 1982 to 2021.

### Supplementary Information

Supplementary Figures.

## Data Availability

Publicly available datasets were analyzed in this study. These data can be found in: OI-SST: https://www.esrl.noaa.gov/psd/data/gridded/; ERA5 and ORAS5: https://cds.climate.copernicus.eu/; SLA and Globcurrent current velocity: http://marine.copernicus.eu/; Moored velocities at 11°S are available at: 10.1594/PANGAEA.962193 and 10.1594/PANGAEA.939249. Shipboard velocities at 11°S during the RV Sonne cruise SO283 are available at: 10.1594/PANGAEA.940306. NPP: http://sites.science.oregonstate.edu/ocean.productivity/index.php.
