# Supplementary material for: Drivers and impact of the 2021 extreme warm event in the tropical Angolan upwelling system
Source: Sci Rep. 2024 Jul 22;14:16824. doi: 10.1038/s41598-024-67569-7 (PMC11263681; doi:10.1038/s41598-024-67569-7)
Supplement: Supplementary file 1 — Supplementary Figures. [file 41598_2024_67569_MOESM1_ESM.docx]

**Supplementary figures**

**
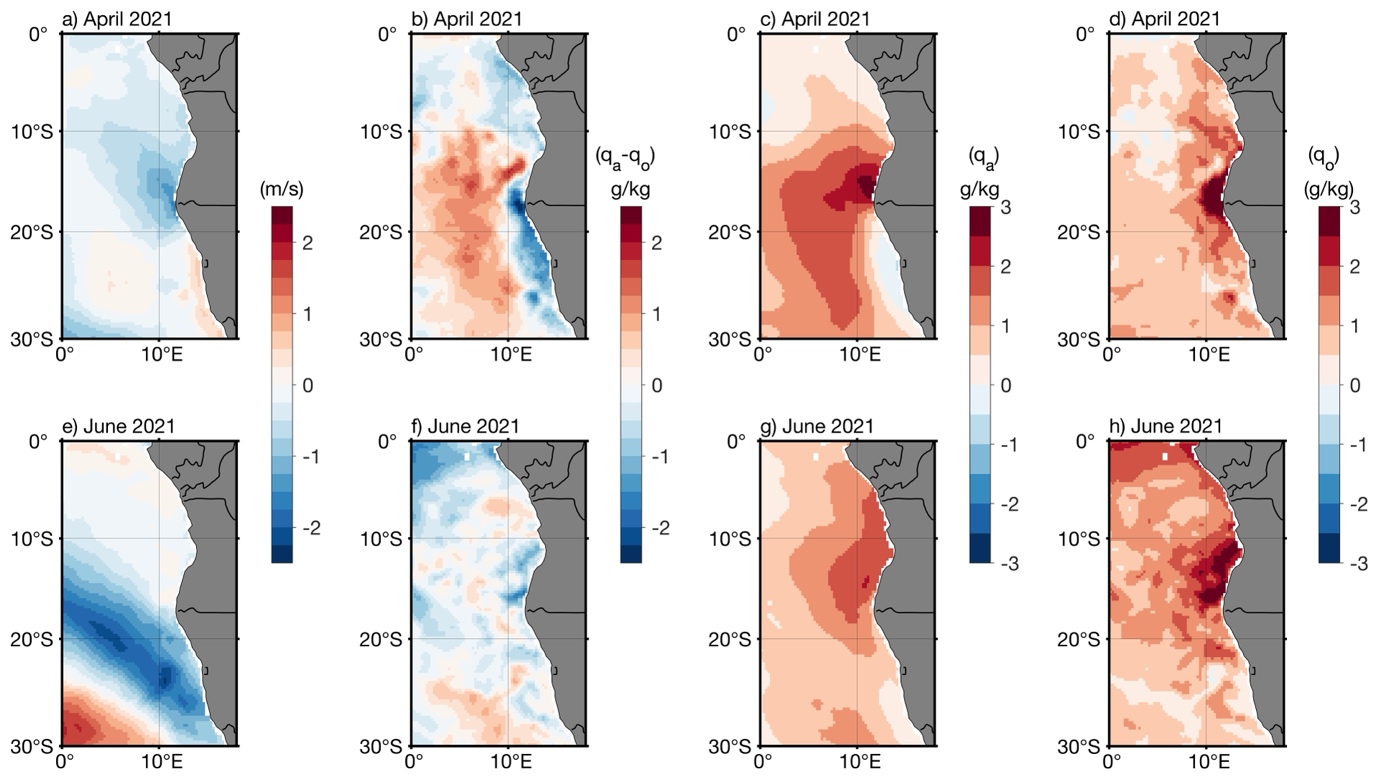
**

Figure S1: Monthly detrended anomalies of: (a) ERA5 surface wind speed, (b) specific humidity difference between the air at 10 m and sea surface; (c) ERA5 specific humidity of air at 10 m; (d) same as (c) but for the specific humidity at the sea surface. (e-h) same as (a-d) but in June 2021.


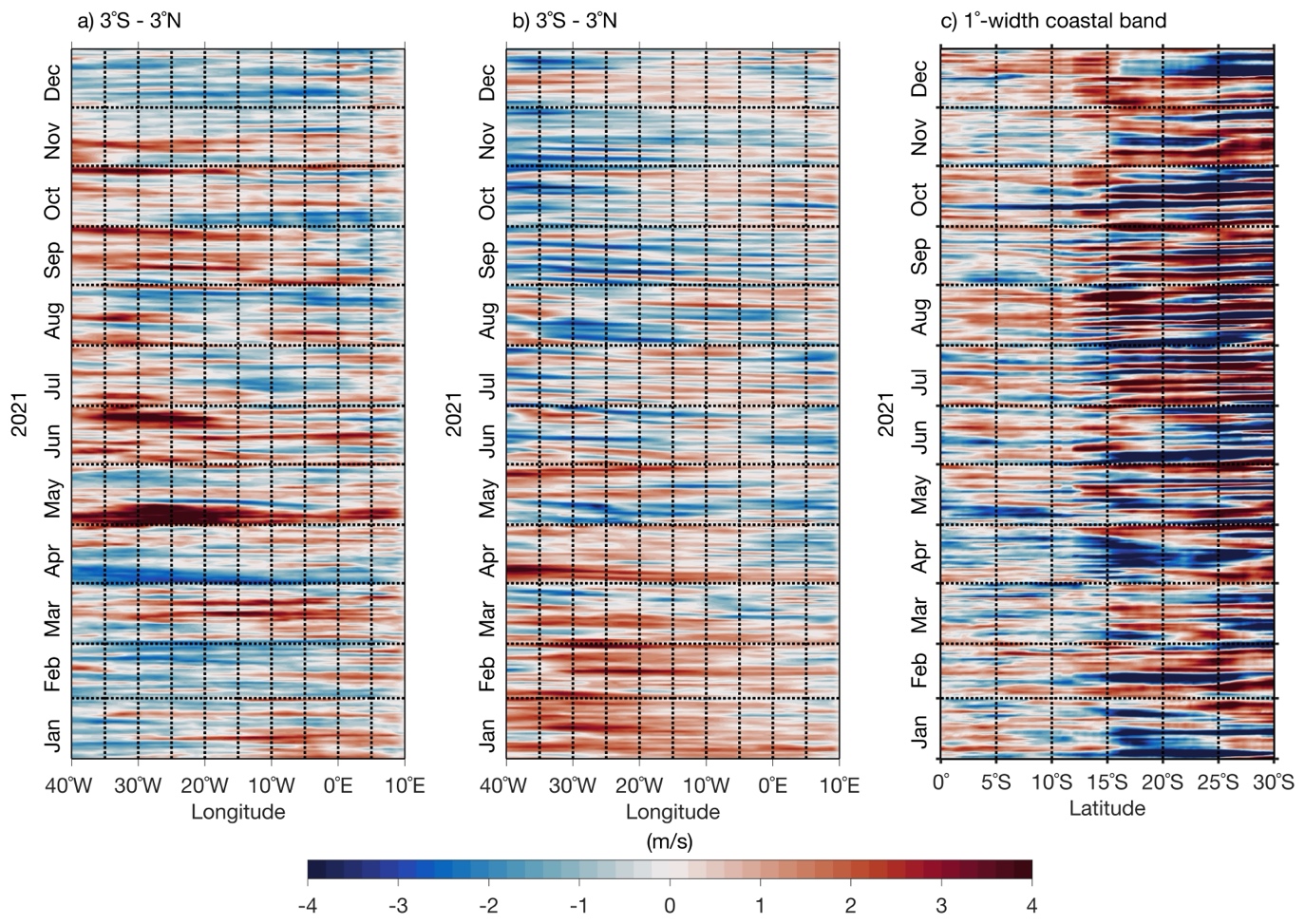


Figure S2: a) Hovmöller diagram of detrended daily ERA5 zonal wind anomalies along the equator (averaged over 3°S – 3°N) from 40°W to the African coastline between January (bottom) to December 2020 (top). (b) Same as (a) but for meridional wind anomalies. c) Hovmöller diagram of detrended daily ERA 5 meridional wind anomalies along the Southern African coast (averaged within 1°-width coastal band) from 0°S to 30°S. The anomalies are calculated relative to the period January 1982 and December 2021.


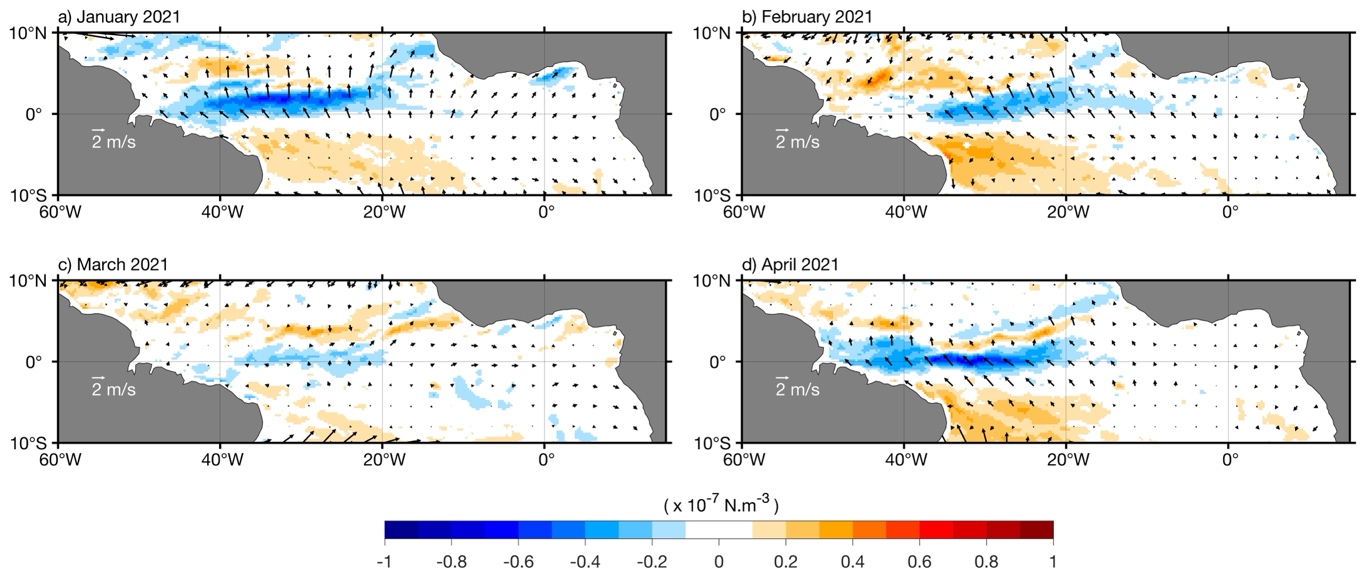


Figure S3: (a-d) Monthly detrended anomalies of ERA5 surface winds (arrows) and derived wind stress curl (shading, 1e-7 N/m^3^) from January to April 2021. The anomalies are calculated relative to the period January 1982 and December 2021.


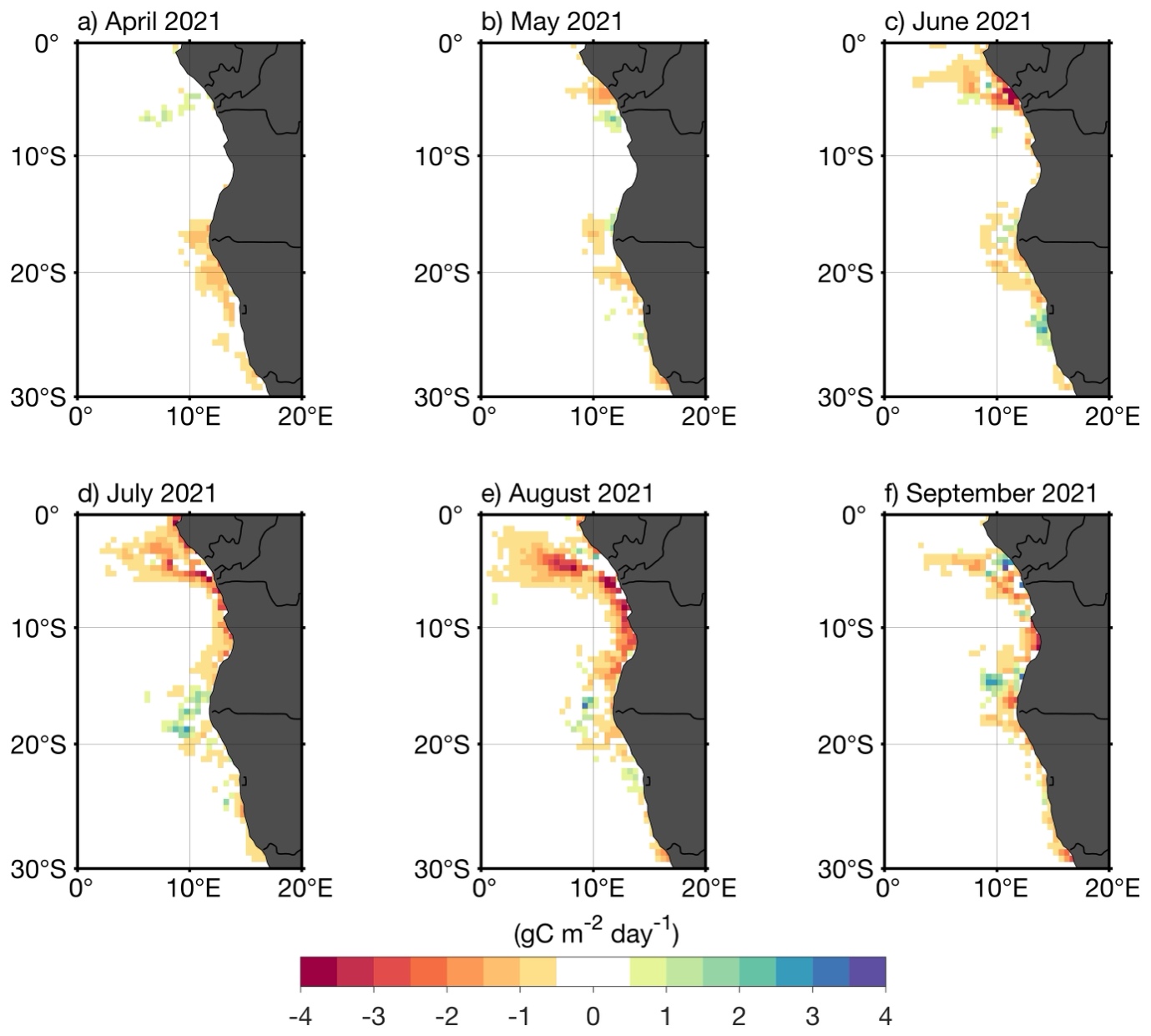


Figure S4: (a-d) Monthly detrended anomalies of NPP from April to September 2021 in the southeastern tropical Atlantic. The anomalies are calculated relative to the period July 2002 and December 2021.


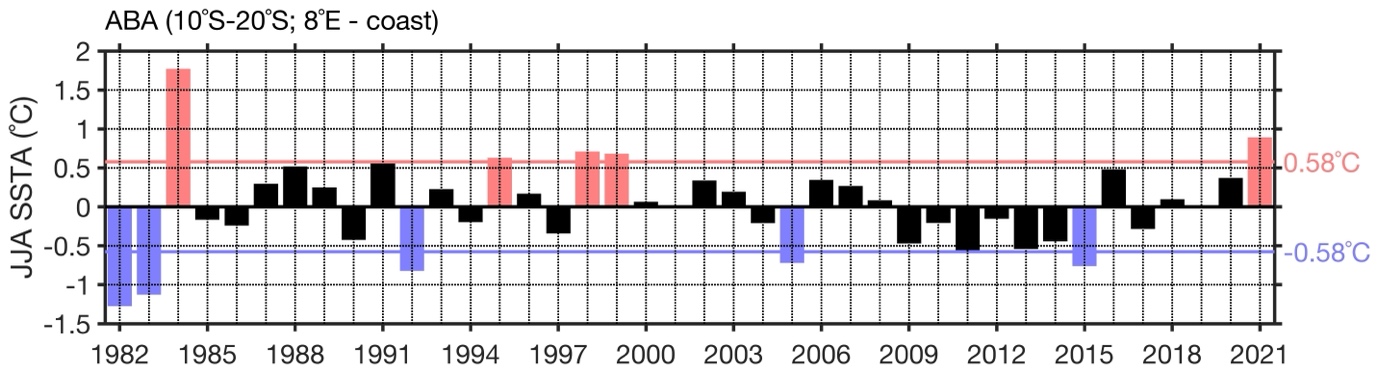


Figure S5: a) Same as Fig. 1a but using HadISST. The anomalies are calculated relative to the period January 1982 and December 2021.


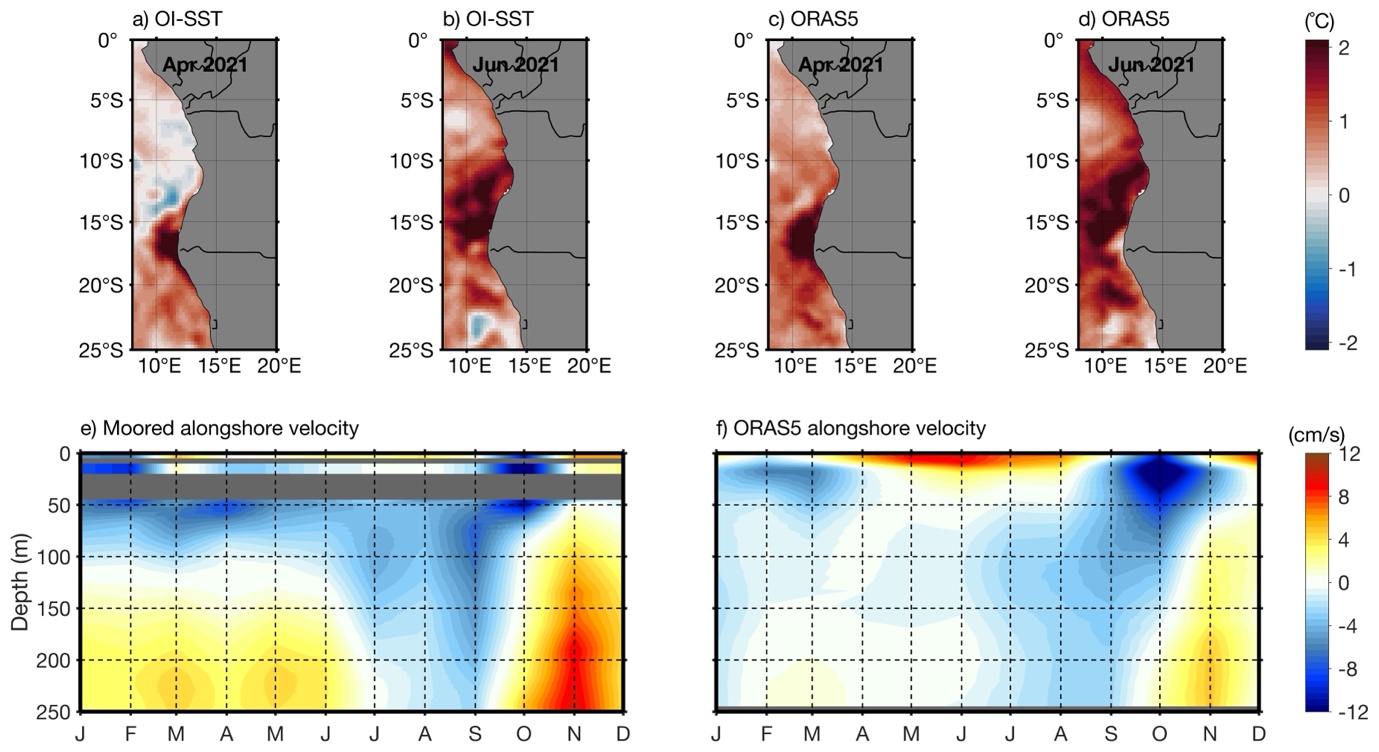


Figure S6: Detrended anomalies of SST in a) April and b) June 2021 for OI-SST. (c-d) same as (a-b) but for ORAS5. The anomalies are calculated relative to the period January 1982 and December 2021. e) Seasonal cycle of alongshore velocities in the upper 15-m depth from Globcurrent and moored alongshore velocities (45-250-m depth) at 13°00’E; 10°50’S between August 2013 and December 2021. f) Seasonal cycle of alongshore velocities from ORAS5 averaged at the closest points around the mooring position between August 2013 and December 2021.
